# The Neurocognitive Basis for Impaired Dual-Task Performance in Senior Fallers

**DOI:** 10.3389/fnagi.2016.00020

**Published:** 2016-02-09

**Authors:** Lindsay S. Nagamatsu, C. Liang Hsu, Michelle W. Voss, Alison Chan, Niousha Bolandzadeh, Todd C. Handy, Peter Graf, B. Lynn Beattie, Teresa Liu-Ambrose

**Affiliations:** ^1^Department of Psychology, University of British ColumbiaVancouver, BC, Canada; ^2^Department of Physical Therapy, University of British ColumbiaVancouver, BC, Canada; ^3^Djavad Mowafaghian Centre for Brain Health, University of British ColumbiaVancouver, BC, Canada; ^4^Department of Psychology, University of IowaIowa City, IA, USA; ^5^Faculty of Medicine, University of British ColumbiaVancouver, BC, Canada; ^6^Division of Geriatric Medicine, Faculty of Medicine, University of British ColumbiaVancouver, BC, Canada

**Keywords:** falls risk, fallers, dual-task, fMRI, aging neuroscience

## Abstract

Falls are a major health-care concern, and while dual-task performance is widely recognized as being impaired in those at-risk for falls, the underlying neurocognitive mechanisms remain unknown. A better understanding of the underlying mechanisms could lead to the refinement and development of behavioral, cognitive, or neuropharmacological interventions for falls prevention. Therefore, we conducted a cross-sectional study with community-dwelling older adults aged 70–80 years with a history of falls (i.e., two or more falls in the past 12 months) or no history of falls (i.e., zero falls in the past 12 months); *n* = 28 per group. We compared functional activation during cognitive-based dual-task performance between fallers and non-fallers using functional magnetic resonance imaging (fMRI). Executive cognitive functioning was assessed via Stroop, Trail Making, and Digit Span. Mobility was assessed via the Timed Up and Go test (TUG). We found that non-fallers exhibited significantly greater functional activation compared with fallers during dual-task performance in key regions responsible for resolving dual-task interference, including precentral, postcentral, and lingual gyri. Further, we report slower reaction times during dual-task performance in fallers and significant correlations between level of functional activation and independent measures of executive cognitive functioning and mobility. Our study is the first neuroimaging study to examine dual-task performance in fallers, and supports the notion that fallers have reduced functional brain activation compared with non-fallers. Given that dual-task performance—and the underlying neural concomitants—appears to be malleable with relevant training, our study serves as a launching point for promising strategies to reduce falls in the future.

## Introduction

Falls is a common geriatric syndrome (Inouye et al., 2007), experienced by approximately 30% of community-dwelling older adults annually, and negatively impact quality of life and functional independance. Impaired cognitive function is now recognized as a key risk factor for falls (Hsu et al., 2012). Importantly, recent research has highlighted that even mild reductions in cognitive abilities can significantly increase falls risk (Anstey et al., 2006).

The relationship between impaired cognitive function and falls is evident in dual-task performance. Specifically, when faced with the challenge of completing two tasks simultaneously, fallers perform significantly worse than their non-falling counterparts (Shumway-Cook et al., 1997; Verghese et al., 2002; Springer et al., 2006; Faulkner et al., 2007; Liu-Ambrose et al., 2009). For example, older adults who stop walking while engaged in conversation are more likely to experience a fall (Lundin-Olsson et al., 1997). Similarly, Verghese et al. (2002) reported that gait speed while concurrently reciting letters of the alphabet was significantly associated with number of falls over a period of 12 months in older adults. Furthermore, we demonstrated that fallers have impaired performance on an ecologically valid virtual-reality dual-task, where they were required to cross a simulated busy street while engaging in a conversation on a hands-free phone (Nagamatsu et al., 2011).

Although there is now an established relationship between dual-task ability and falls risk, research on the underlying neural concomitants for this association has been limited because there are few published neuroimaging studies comparing fallers and non-fallers—yet such knowledge has the potential to lead to the refinement and development of behavioral, cognitive, or neuropharmacological interventions for falls prevention. The only previous functional neuroimaging studies of fallers found altered brain activation compared to non-fallers (Liu-Ambrose et al., 2008; Nagamatsu et al., 2013a; Hsu et al., 2014). Specifically, during performance of an executive cognitive task that engages selective attention and conflict resolution, older adults with a history of recent falls exhibited reduced blood-oxygen-level dependent (BOLD) activation in key brain regions, including the cerebellum (Liu-Ambrose et al., 2008) and the medial frontal gyrus (Nagamatsu et al., 2013a), areas critical for motor planning and the integration of higher order cognitive processing (Bellebaum and Daum, 2007; Timmann and Daum, 2007; Boyd et al., 2009). Although differences in activation have been identified between fallers and non-fallers in the context of executive cognitive functioning, examining functional activation during dual-tasking is novel because the performance of two tasks concurrently employs additional brain regions not responsible for performing either single task alone (D’Esposito et al., 1995).

The primary aim of our study was to determine differences in activation between fallers and non-fallers during dual-task performance. Our secondary aims were to determine whether these differences relate to behavioral task performance, and whether functional activation relates to independent measures of executive cognition and/or mobility. To examine the above aims, we conducted a cross-sectional study of older adults with and without a history of falls. Aligning with our previous findings (Liu-Ambrose et al., 2008; Nagamatsu et al., 2013a), we hypothesized that fallers would show reduced functional activation during dual-task performance, and that functional activation would be associated with secondary measures of executive cognition and mobility.

## Materials and Methods

### Participants

Fifty-six community-dwelling men and women aged 70–80 years were recruited from local newspaper advertisements. Participants were eligible if they: (1) scored ≥24/30 on the Mini-Mental Status Examination (MMSE); (2) were right hand dominant, as measured by the Edinburgh Handedness Inventory; (3) were living independently in their own home; and (4) had visual acuity of at least 20/40, with or without corrective lenses. We excluded those who: (1) had a neurodegenerative disease, stroke, dementia (of any type), or psychiatric condition; (2) had clinically significant peripheral neuropathy or severe musculoskeletal or joint disease; (3) were taking psychotropic medications; (4) had a history indicative of carotid sinus sensitivity; (5) were living in a nursing home, extended care facility, or assisted-care facility; or (6) did not meet MRI scanning requirements. Based on their falls history in the 12 months prior to study entry, participants were categorized as “fallers” or “non-fallers”. Fallers were defined as those who reported ≥2 minimal displacement, non-syncopal falls in the past 12 months (Holtzer et al., 2007). Non-fallers were defined as those who reported 0 falls in the past 12 months. Falls were indicated via self-report and corroborated by a friend or family member to reduce the risk of recall bias. All participants completed two separate sessions: (1) an assessment session to collect descriptive measures; and (2) functional magnetic resonance imaging (fMRI) scanning. Ethics approval was obtained from the Vancouver Coastal Research Health Institute and University of British Columbia’s Clinical Research Ethics Board. All participants provided written informed consent.

### Descriptive Variables

General cognitive status was ascertained using the Montreal Cognitive Assessment (MoCA; Nasreddine et al., 2005) and MMSE (Folstein et al., 1975). Depression was screened using the 15-item Geriatric Depression Scale (GDS; Yesavage, 1988). Number of comorbidities was obtained using the Functional Comorbidity Index (FCI; Groll et al., 2005). We assessed physiological falls risk using the valid and reliable Physiological Profile Assessment (PPA; Lord et al., 2003), which takes into account five separate physiological measures to compute a standardized falls risk score. A *z*-score of 0–1 indicates mild falls risk, 1–2 indicates moderate falls risk, and above two indicates marked falls risk. Falls-related self-efficacy was reported using the Activities-Specific Balance Confidence scale (ABC; Powell and Myers, 1995). General mobility was assessed using the Timed Up and Go test (TUG; Podsiadlo and Richardson, 1991). Participants were required to stand from a seated position, walk a distance of three meters, return to their seat, and sit back down. The average time to complete two independent trials was recorded, with shorter times indicating better mobility.

### Executive Functions

We assessed three separate executive functions (Miyake et al., 2000) using pen and paper tests. The protocol we followed has been previously reported (Hsu et al., 2014). Briefly, we used the Stroop Color Word Test to assess selective attention and conflict resolution (Stroop, 1935). The completion time difference between the incongruent condition (i.e., name the ink color in which color words were printed) and the neutral condition (i.e., read out the color of colored “X’s”) was calculated, with smaller times indicating better performance. Set-shifting was assessed using the Trail Making Test Parts A and B (Gaudino et al., 1995). The difference in time to complete each part was calculated, with smaller difference scores indicating better performance. Digits Forward and Backward were used to assess working memory (Wechsler, 1981). The difference between the forward and backward test scores were calculated, with smaller scores indicating better working memory.

### Dual-Task Performance

**Figure 1:**
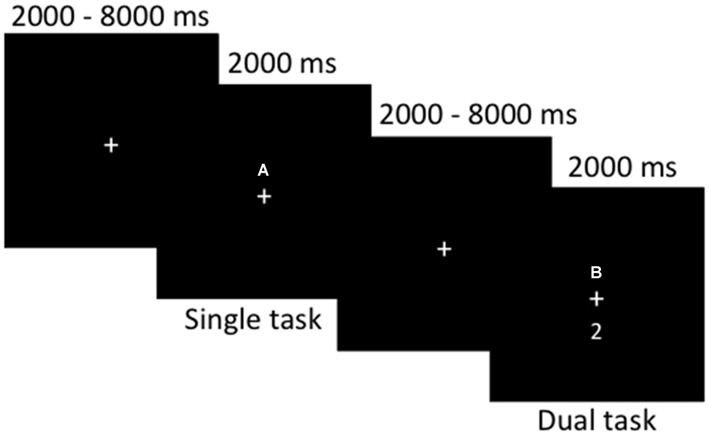
**Stimulus timing and presentation for the dual-task paradigm during functional magnetic resonance imaging (fMRI) scanning.** Single- or dual-task stimuli were presented for a fixed duration of 2000 ms, with 2000–8000 ms of fixation randomly jittered between each trial. Participants completed a total of 80 event-related trials.

Participants completed a computerized dual-task paradigm in the MRI scanner (for stimulus presentation and timing, see Figure 1). Participants were presented at fixation with either a single number or letter (single-task) or a number and letter concurrently (dual-task). Participants were instructed to make a two-choice discrimination for numbers (2 or 3) and/or letters (A or B) by responding as quickly and accurately as possible via button presses with their left hand for letters and right hand for numbers (index fingers corresponding to “B” and “2” and middle fingers corresponding to “A” and “3”, respectively). Each participant completed 40 single-task trials and 40 dual-task trials, with 82 fixation (null) trials; trial type was inter-mixed in an event-related design, randomized for each subject. Reaction times and accuracy were recorded. Participants completed the task during one continuous fMRI scan lasting approximately 11 min. Prior to MRI scanning, all participants completed a practice block outside the scanner to become familiar with the task.

### fMRI Data Acquisition

Scanning was completed at the UBC Hospital using a 3.0T Intera Achieva MRI scanner (Philips Medical Systems Canada, Markham, Ontario, Canada). Transverse echo-planar imaging (EPI) images in-plane with the anterior and posterior commissure (AC-PC) line were acquired using a gradient-echo pulse sequence and sequential slice acquisition (repetition time, TR = 2000 ms, echo time, TE = 30 ms, flip angle = 90°, 36 contiguous slices at 3 mm skip 1 mm, in-plane resolution of 128 × 128 pixels reconstructed in a field of view of 240 mm). Each functional run began with four TR’s during which no data were acquired to allow for steady-state tissue magnetization. A total of 330 EPI volumes were collected in each functional run. High-resolution, T1-weighted axial images were also taken of each participant (TR = 8 ms, TE = 3.7 ms, bandwidth = 2.26 kHz, voxel size = 1 mm^3^).

### fMRI Preprocessing and Analysis

Data were preprocessed using FEAT (version 5.98) within FMRIB’s FSL (version 4.1.4). During first-level preprocessing, individual participant data was motion corrected (Jenkinson, 2003) and spatially-smoothed with a gaussian kernel of 5.0 mm full width at half maximum. The resulting task model was then convolved using a double-gamma function to fit the task-evoked BOLD response. Registration was completed using a two-step process in FLIRT, where each participant’s fMRI images were registered to subject-specific high-resolution T1 images, which were then registered to standard space (Jenkinson and Smith, 2001). Contrasts were set up to examine activation during both single- and dual-task conditions and the difference between the conditions (i.e., single > dual; dual > single). The parameter estimate maps and variance maps for the above contrasts were then forwarded to a higher-level mixed-effects analysis, where data from all participants were combined at the group level. We used FLAME in FSL to accurately model and estimate between-group differences. Statistically significant clusters of activation that differed as a function of falls status were identified examining the statistical maps generated by the fallers > non-fallers and non-fallers > fallers contrasts, using a voxel-wise threshold of *z* > 1.65, *p* < 0.05 combined with a cluster probability threshold of *p* < 0.05 to account for multiple comparisons and reduce type 1 error rates (Worsley et al., 1992). For clusters showing significant between-group differences in activation, we report the most probable anatomical label using the Harvard-Oxford Cortical Structural Atlas packaged in FSL. For each region of interest (ROI) identified, we created spherical ROIs centered on the Montreal Neurological Institute (MNI) coordinates of the peak location, equaling approximately 125 anatomical voxels. For each ROI, percent signal change was extracted for each participant individually using Featquery within FSL. The percent signal changes were the imported into SPSS (Version 21 for Mac) for further analysis.

### Additional Statistical Analyses

Descriptive and behavioral data were analyzed by independent samples *t*-tests to examine between-group differences. We calculated Pearson Product Moment correlations to examine associations between functional activation, as indicated by individual-level percent signal changes for the above ROIs (i.e., those significantly different between fallers and non-fallers), and measures of behavioral performance, executive cognition, and mobility. Outliers, defined as scores that were greater than three standard deviations from the mean, were removed from analyses; such cases are specified below (i.e., one case for each of Stroop and TUG).

## Results

### Participants

Demographic information for participants as a function of falls status is presented in Table 1. Fallers had significantly more comorbidities than non-fallers, *t*_(54)_ = 1.97, *p* = 0.05. We examined whether the inclusion of FCI as a covariate in our fMRI and correlational analyses would alter our results; we did not find this to be the case, and therefore, FCI was not included as a covariate in the results presented below. Otherwise, there were no significant differences between our two groups for the remaining descriptive variables, include MMSE, MoCA, and measures of mobility (all *p*’s > 0.09). There were no between-group differences on performance for the three measures of executive cognitive functioning (all *p*’s > 0.50).

**Table 1:** **Demographic characteristics and behavioral performance of sample**.

Variable	Fallers (*n* = 28) Mean (*SD*)	Non-fallers (*n* = 28) Mean (*SD*)
Age, years	75.29 (3.35)	75.11 (2.83)
Sex, count (%) female	21 (77)	18 (64)
Education, count (%)
Less than high school diploma	4 (14.3)	3 (10.7)
High school diploma	5 (17.9)	3 (10.7)
Some university without diploma	2 (7.1)	1 (3.6)
Trades or professional certificate	5 (17.9)	3 (10.7)
University diploma or degree	12 (42.8)	18 (64.3)
Geriatric depression scale	0.64 (1.57)	0.39 (1.07)
Functional comorbidity index	3.21 (1.71)	2.36 (1.54)*
Mini-mental status examination	28.21 (1.55)	28.29 (1.61)
Montreal cognitive assessment	24.29 (3.09)	24.82 (3.29)
Activities-specific balance confidence	82.36 (17.66)	89.21 (10.82)
Physiological profile assessment	0.42 (0.77)	0.14 (0.77)
Timed up and go, s	8.23 (3.67)	7.21 (1.10)
Trail making test (Part B–Part A), s	47.38 (33.83)	52.33 (44.42)
Stroop color word test (Part 3–Part 2), s	52.77 (23.37)	52.00 (25.64)
Digit span (Forward–Backward), No.	3.71 (1.76)	4.07 (2.32)
Single-task reaction time^a^	1073.46 (117.25)	1046.71 (115.15)
Dual-task reaction time^a^	1363.57 (85.25)	1302.67 (94.70)**
Single-task errors^b^	8.86 (8.78)	7.46 (5.73)
Dual-task errors^b^	18.75 (7.63)	18.04 (9.65)

^a^ms; includes correct trials only. ^b^Number of incorrect responses (out of 40). *p ≤ 0.05; **p ≤ 0.01.

### Dual-Task Performance

Behavioral performance for fallers and non-fallers are presented in Table 1. Reaction times were calculated for correct responses for single- and dual-task trials separately; dual-task reaction time was calculated based on the average time to respond to each stimulus. There were no significant differences between fallers and non-fallers for the single-task condition (*p* = 0.39). For the dual-task condition, fallers responded significantly slower than non-fallers, *t*_(54)_ = 2.53, *p* = 0.01. For accuracy, there were no between-group differences, *p* = 0.49 and 0.76 for number of incorrect trials during single- and dual-task trials, respectively.

### fMRI Results

**Figure 2:**
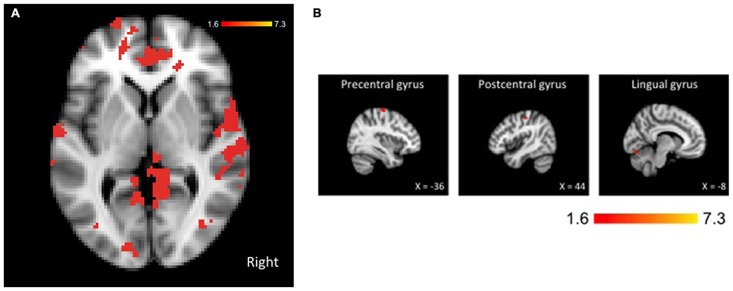
**Brain activation for the dual-task > single-task contrast showing greater activation for non-fallers compared with fallers.** The threshold was set at *p* < 0.05, presented in neurological orientation. **(A)** Whole brain activation shown at coordinates *x* = 0, *y* = 0, *z* = 0. **(B)** Three significant clusters with maxima in precentral, postcentral, and lingual gyri. The threshold was set at *p* < 0.05, presented in neurological orientation.

Comparing our two groups, we identified three clusters—the left precentral and lingual gyri and the right post-central gyrus—that were significantly more active in non-fallers to fallers for the dual-task > single-task contrast, as seen in Figure 2. Furthermore, there were 15 local maxima that were identified for the same contrast, as seen in Table 2. These regions included bilateral pre- and post-central gyri, lingual gyrus, and occipital fusiform gyrus, left frontal pole, supplementary motor cortex, and cerebellum, and right central and parietal operculum. There were no regions that were more active for fallers compared with non-fallers.

**Table 2:** **Significant clusters for non-fallers > fallers, dual-task > single-task**.

Hemisphere	Area	MNI coordinates			*Z*	Voxels
		*X*	*Y*	*Z*		
Left	Precentral gyrus	−36	−16	64	4.26	14482
Right	Lingual/posterior cingulate gyrus	10	−50	2	3.83	
Left	Frontal pole	−30	58	22	3.57	
Left	Post-central gyrus	−50	−18	56	3.53	
Left	Supplementary motor cortex	−4	−4	70	3.37	
Right	Post-central gyrus	44	−22	50	3.31	3117
Right	Precentral gyrus	20	−24	70	3.29	
Right	Precentral gyrus/postcentral gyrus	42	−20	56	3.26	
Right	Central opercular cortex	58	2	4	3.25	
Right	Parietal operculum cortex	60	−26	18	3.10	
Left	Lingual gyrus	−8	−82	−14	3.81	2025
Left	Occipical fusiform/lingual gyrus	−12	−80	−16	3.57	
Left	Occipital fusiform gyrus	−26	−82	−16	3.36	
Right	Occipital fusiform gyrus	30	−76	−14	3.00	
Left	Cerebellum	−4	−72	−16	2.85	

Our above contrast of interest, dual-task > single-task does not allow us to speak to whether the differences observed between fallers and non-fallers are due to underlying differences in activation during single-task performance, dual-task performance, or a combination of both. Thus, we examined activation for each task-type separately for the ROIs determined above (see Table 3). Overall, we found that during both single- and dual-task processing, non-fallers exhibited significantly greater activation than fallers. Specifically, for single-task processing, this activation was demonstrated in bilateral precuneous extending towards the lateral occipital cortex, left post-central and posterior cingulate gyri. For dual-task processing, non-fallers also showed significantly greater activation than fallers in left precentral gyrus, posterior temporal fusiform cortex, frontal pole, and posterior supramarginal gyrus, as well as the right posterior cingulate gyrus. In contrast, there were no regions that were significantly more active for fallers compared with non-fallers during either task condition.

**Table 3:** **Significant clusters for non-fallers > fallers, single-task and dual-task separately**.

Hemisphere	Area	MNI coordinates			*Z*	Voxels
		*X*	*Y*	*Z*		
**Single-task**
Left	Precuneous cortex/lateral occipital cortex, superior	−6	−66	62	3.25	1587
Left	Postcentral gyrus	−4	−50	78	3.24	
Right	Precuneous cortex	2	−68	56	3.07	
Right	Lateral occipital cortex, superior	20	−74	62	3.00	
Left	Cingulate gyrus, posterior	−10	−20	42	2.90	
**Dual-task**
Left	Precentral gyrus	−36	−18	64	4.41	39599
Left	Temporal fusiform cortex, posterior	−34	−42	−20	4.35	
Left	Frontal pole	−30	58	22	4.24	
Right	Cingulate gyrus, posterior	8	−46	2	4.21	
Left	Supramarginal gyrus, posterior	−64	−42	30	4.01	

### Correlations

First, functional activation during fMRI was not significantly associated with dual-task behavioral performance, all *p*’s > 0.05. Second, examining the association between functional activation and our independent measures of executive cognition assessed outside the scanner, greater activation was associated with better working memory and response inhibition. Specifically, higher activation in left cerebellum and bilateral occipital fusiform gyri were significantly associated with better Digit Span scores, *r*_(56)_ = −0.32, *p* < 0.02 (left cerebellum), *r*_(56)_ = −0.27, *p* < 0.05 (left occipital fusiform gyrus), and *r*_(56)_ = −0.28, *p* < 0.04 (right occipital fusiform gyrus). In addition, higher activation in right pre-/post-central gyrus was significantly associated with less interference on the Stroop test, *r*_(54)_ = −0.28, *p* = 0.04 (one outlier for Stroop removed *z* = 3.30). Third, examining the associations between functional activation and mobility measures revealed that greater activation was also associated with better mobility. This was demonstrated by significant correlations between TUG performance and activation in left occipital fusiform gyrus, *r*_(55)_ = −0.27, *p* = 0.04, left supplementary motor cortex, *r*_(55)_ = −0.31, *p* = 0.02, and right lingual/posterior cingulate gyrus, *r*_(55)_ = −0.33, *p* = 0.01 (one outlier for TUG removed *z* = 5.91).

## Discussion

In this cross-sectional study examining differences in dual-task performance and underlying neural correlates between fallers and non-fallers, we report a network of regions that are significantly more active in non-fallers compared with fallers; these fMRI results were combined with slower reaction times during dual-task performance in fallers. Taken together, our current study complements our previous findings that fallers have reduced functional activation during performance of a cognitive task (Liu-Ambrose et al., 2008; Nagamatsu et al., 2013a) and provides the first evidence that underlying neural differences may be responsible for altered dual-task performance in fallers. Several noteworthy points of discussion follow.

First, we now have collective evidence that fallers have overall reductions in BOLD activation during cognitive tasks. Adding to previous findings (Liu-Ambrose et al., 2008; Nagamatsu et al., 2013a), our current study found greater activation in non-fallers compared with fallers—not only on the dual-task > single-task contrast, but for each task-type independently as well. It has been proposed that reduced BOLD signals may be a consequence of disrupted functional and/or structural integrity of the brain. Indeed, neural changes that are inherent to pathological aging can alter neurovascular coupling, which is ultimately responsible for the BOLD response (D’Esposito et al., 2003). Of central importance to our findings reported here, impaired mobility has been linked to structural abnormalities in the brain, including increased number of white matter hyperintensities (Zheng et al., 2012) and lesions (Zheng et al., 2011), in addition to altered functional connectivity (Hsu et al., 2014). This idea is further substantiated by the significant correlations that we have reported—that higher functional activation is associated with both better executive functioning and mobility. Thus, our study supports the current prevailing hypothesis that mobility, cognitive function, and structural and functional brain integrity are closely interconnected.

Second, the specific brain regions that we found to have higher activation in non-fallers compared to fallers during dual task performance map onto regions that have previously been found to activate during the performance of a similar two-choice reaction time dual-task test, including precentral, postcentral, and lingual gyri (Schubert and Szameitat, 2003). Together, these regions appear to support the cognitive coordination required to respond to two stimuli simultaneously, above and beyond processing and response to a single stimulus. One region in particular—the right operculum—has been found to be critical for resolving dual-task interference when attention is simultaneously divided between two perceptual attention processes (Jiang, 2004). This concurs with previous reports that visual-spatial attention to both task-relevant and task-irrelevant information may be disrupted in fallers at the perceptual-level, as indicated by reduced P1 and N1 event-related potential (ERP) component amplitudes (Nagamatsu et al., 2009, 2013b). Importantly, a processing bottleneck at the perceptual attention-level may account for the differences observed between fallers and non-fallers later during the processing stream that we report here, including the fusiform gyrus, pre- and post-central gyri, and frontal pole (Goodale and Milner, 1992). This hypothesis, however, remains speculative at this point; future studies to support the idea that fallers experience an early perceptual disadvantage are warranted.

Finally, the ultimate question we want to ask is whether the results of our study might extend to real-world dual-task performance in fallers? The computer-based dual-task paradigm used in the present study was also previously used in a study examining behavioral dual-task performance in older adults at-risk for falls in “real-life” circumstances (Nagamatsu et al., 2011). In addition to the computer-based task, these participants also crossed a simulated busy street in a virtual environment by walking on a manual treadmill either alone (single-task) or while concurrently talking on a hands-free phone (dual-task). Those at-risk for falls, as determined via the PPA, had significantly poorer street-crossing performance compared with those not-at-risk for falls. Notably, performance in the virtual environment was significantly correlated with performance on the computer-based dual-task paradigm. Thus, although our task only involved a computer-based task, it has previously been shown to be related to performance in the real world. Interestingly, previous work has found that dual-task ability can be successfully improved in older adults through a training program (Erickson et al., 2007)—and that such benefits correspond to changes in neural activation (Erickson et al., 2007) and extend to non-trained dual-tasks as well (Bherer et al., 2005). Therefore, cognitive training may provide a novel intervention strategy for improving dual-task ability and reducing falls risk among this at-risk population.

Our cross-sectional study is among the first to examine functional differences in brain activation between fallers and non-fallers. However, we note the limitations of our current study. For one, the dual-task paradigm we used was strictly cognitive—that is, participants performed two cognitive tasks simultaneously. This was due to the constraint of having the task performed in the MRI scanner where movement must be eliminated. In contrast, the majority of dual-task studies on fallers have involved combining a cognitive and physical task. Nevertheless, as mentioned above, the paradigm that was used has been shown to be positively associated with real-life dual-task performance (Nagamatsu et al., 2011). Second, although we demonstrated that fallers had both reduced functional activation and slower reaction times during dual-task performance, activation level and behavioral performance were not significantly correlated. This seemingly disparate finding may be reconciled by the fact that these two reflect separate processes—namely, cognitive processing of stimuli vs. manual response output. Another limitation is that our cross-sectional study design does not allow us to infer causality between falls and impaired dual-task performance and/or altered functional brain activation. Future prospective studies are therefore warranted. Lastly, we recognize that our sample is relatively homogeneous, including healthy and high functioning older community-dwelling adults; thus future studies to examine how our findings may apply to adults who are older or younger than our sample, or with various cognitive and/or functional statuses are encouraged.

## Conclusion

In conclusion, we present the first neuroimaging study to examine differences between fallers and non-fallers during dual-task performance. In addition to supporting the notion that fallers have reduced functional brain activation compared with non-fallers, our results point towards the possibility that such impairments may arise at the perceptual processing stage. Importantly, such failures to attend to relevant stimuli would provide fallers with a marked disadvantage when navigating the environment. Given that dual-task performance—and the underlying neural concomitants—appear to be malleable with relevant training, our study serves as a launching point for promising opportunities to reduce falls risk in the future.

## Author Contributions

LSN: Study conception and design, analysis and interpretation of data, and manuscript preparation; CLH, AC, and NB: Acquisition of data, data analysis, and critical review of manuscript; MWV, TCH, PG, BLB: Study conception and design, interpretation of data, and critical review of manuscript; TL-A: Study conception and design, analysis and interpretation of data, and manuscript preparation. All authors approve this version of the manuscript and are accountable for all aspects of the work.

## Conflict of Interest Statement

The authors declare that the research was conducted in the absence of any commercial or financial relationships that could be construed as a potential conflict of interest.
